# Combining Hypermethylated *RASSF1A* Detection Using ddPCR with miR-371a-3p Testing: An Improved Panel of Liquid Biopsy Biomarkers for Testicular Germ Cell Tumor Patients

**DOI:** 10.3390/cancers13205228

**Published:** 2021-10-18

**Authors:** João Lobo, Lieke M. J. van Zogchel, Mohammed G. Nuru, Ad J. M. Gillis, C. Ellen van der Schoot, Godelieve A. M. Tytgat, Leendert H. J. Looijenga

**Affiliations:** 1 Princess Máxima Center for Pediatric Oncology, Heidelberglaan 25, 3584 CS Utrecht, The Netherlands; jpedro.lobo@ipoporto.min-saude.pt (J.L.); L.M.J.vanZogchel@prinsesmaximacentrum.nl (L.M.J.v.Z.); A.J.M.Gillis@prinsesmaximacentrum.nl (A.J.M.G.); 2 Cancer Biology and Epigenetics Group, Research Center of IPO Porto (CI-IPOP)/RISE@CI-IPOP (Health Research Network), Portuguese Oncology Institute of Porto (IPO Porto)/Porto Comprehensive Cancer Center (Porto.CCC), R. Dr. António Bernardino de Almeida, 4200-072 Porto, Portugal; 3 Department of Pathology, Portuguese Oncology Institute of Porto (IPOP), R. Dr. António Bernardino de Almeida, 4200-072 Porto, Portugal; 4 Department of Pathology and Molecular Immunology, ICBAS—School of Medicine and Biomedical Sciences, University of Porto (ICBAS-UP), Rua Jorge Viterbo Ferreira 228, 4050-513 Porto, Portugal; 5 Department of Experimental Immunohematology, Sanquin Research Amsterdam, Plesmanlaan 125, 1066 CX Amsterdam, The Netherlands; karennuru1984@gmail.com (M.G.N.); e.vanderschoot@sanquin.nl (C.E.v.d.S.); 6 Lab. for Exp. Patho-Oncology (LEPO), Department of Pathology, Erasmus MC-University Medical Center, Doctor Molewaterplein 40, 3015 GD Rotterdam, The Netherlands

**Keywords:** germ cell tumors, droplet digital PCR, microRNAs, methylation, biomarkers, miR-371a-3p, *RASSF1A*

## Abstract

**Simple Summary:**

Testicular germ cell tumors are the most common solid cancers in men aged between 15–39 years. There is a need of non-invasive biomarkers for diagnosis and follow-up of these patients. miR-371a-3p has emerged as the most reliable biomarker of this disease, but fails to detect the teratoma subtype, which has clinical implications. In this work we describe a new method that combines miR-371a-3p quantification using RT-qPCR and hypermethylated *RASSF1A* quantification by droplet digital PCR in serum samples of these patients. The combination of both biomarkers detected disease (including teratoma) with a sensitivity of 100%, using cutoffs that made all healthy participants negative, being of interest for implementation in the clinical setting.

**Abstract:**

The classical serum tumor markers used routinely in the management of testicular germ cell tumor (TGCT) patients—alpha fetoprotein (AFP) and human chorionic gonadotropin (HCG)—show important limitations. miR-371a-3p is the most recent promising biomarker for TGCTs, but it is not sufficiently informative for detection of teratoma, which is therapeutically relevant. We aimed to test the feasibility of hypermethylated *RASSF1A* (*RASSF1A_M_*) detected in circulating cell-free DNA as a non-invasive diagnostic marker of testicular germ cell tumors, combined with miR-371a-3p. A total of 109 serum samples of patients and 29 sera of healthy young adult males were included, along with representative cell lines and tumor tissue samples. We describe a novel droplet digital polymerase chain reaction (ddPCR) method for quantitatively assessing *RASSF1A_M_* in liquid biopsies. Both miR-371a-3p (sensitivity = 85.7%) and *RASSF1A_M_* (sensitivity = 86.7%) outperformed the combination of AFP and HCG (sensitivity = 65.5%) for TGCT diagnosis. *RASSF1A_M_* detected 88% of teratomas. In this representative cohort, 14 cases were negative for miR-371a-3p, all of which were detected by *RASSF1A_M_*, resulting in a combined sensitivity of 100%. We have described a highly sensitive and specific panel of biomarkers for TGCT patients, to be validated in the context of patient follow-up and detection of minimal residual disease.

## 1. Introduction

Liquid biopsies represent valuable tools for non-invasively investigating cancer in several settings, including early (differential) diagnosis, follow-up, prediction of prognosis and for guiding treatment decisions [1]. In the last decades, great effort has been made to find and validate optimal biomarkers that can assist in clinical decision-making in oncology [2,3], including microRNAs and methylation of several gene promoters [4,5,6]. More accurate techniques for detection and quantification (including droplet digital polymerase chain reaction (ddPCR)) have increased the interest of translating these biomarkers into the clinical setting [7].

Germ cell tumors (GCTs) are among the most common solid neoplasms in young adult Caucasian men, and the incidence is increasing [8]. GCTs are very diverse, as they recapitulate the various steps of embryonic and germ cell development [9,10]. Because of the prevalence and clinical relevance, GCTs of the testis (TGCTs) are the focus of most studies, including those on liquid biopsies. These tumors are derived from a common precursor (germ cell neoplasia in situ—GCNIS) and are classified into two major classes, the seminomas (SE) and non-seminomas (NS) [11,12]. A definitive diagnosis of TGCTs can only be done after histological assessment of the orchiectomy specimen, hence the need for non-invasive liquid biopsy biomarkers. For these tumors, the major novelty in the liquid biopsy field has been the miR-371a-3p. This microRNA performance has proven superior in diagnostic and follow-up settings when compared with the classical available biomarkers used in the clinic—alpha fetoprotein (AFP), human chorionic gonadotropin subunit beta (β-HCG) and lactate dehydrogenase (LDH). miR-371a-3p has emerged as the most remarkable non-invasive biomarker for diagnosis and follow-up of TGCT patients, as demonstrated by several retrospective and also prospective works by various groups [13,14,15,16,17] (for a recent review see [18]). Its introduction in the clinic is to occur soon and may reduce costs in patient follow-up [19]. However, its major limitation is the inability to detect a subset of NS, which is teratoma (TE) [17,20,21,22]. This leaves a clinically relevant gap in the field of GCTs, since TE represents chemo-resistant disease and needs to be distinguished from fibrosis/necrosis and viable tumor, since for TE surgical excision is the only therapeutic option. However, this surgery is not without risks, both immediate as well as longer term, and classical markers are generally not useful for confirming the presence of this tumor subtype [23,24].

Promoter hypermethylation of the tumor suppressor gene *RASSF1A* is a common feature detected among several pediatric (and non-pediatric) solid malignancies [25,26]. *RASSF1A* seems to function as an epigenetic sensor, although the exact biology is still a matter of debate [27]. *RASSF1A* is viewed as a prototype of a tumor suppressor gene, being inactivated in many malignancies. It interacts with several different pathways (related to cell cycle regulation, metabolism, cell growth, invasion and migration and apoptosis, among many others), which explains its almost universal role as a prognostic biomarker (associated with disease progression and metastasis) and frequently influencing response to treatment [28]. In TGCTs, hypermethylation of *RASSF1A* (*RASSF1A_M_*) has been reported in few studies using tumor tissue, which also demonstrated *RASSF1A_M_* in teratoma, but was only detected in 47% of TGCT liquid biopsy samples [29,30,31]. In line with this, and given the usefulness of this marker for detecting minimal residual disease in blood or bone marrow in several pediatric/young adult tumors [32], we hypothesized that *RASSF1A_M_* testing could also assist in the non-invasive diagnosis of GCTs, fulfilling the clinical need of TE detection. The improvement of the sensitivity of the available molecular assays might also be instrumental in this context.

In this work we used a representative cohort of TGCT patients, to test the suitability of *RASSF1A_M_* detection in circulating cell-free DNA (cfDNA) as a non-invasive biomarker of (T)GCTs at time of diagnosis, as single marker, combined with miR-371a-3p and compared with the classical serum markers. We aim to design a highly sensitive and specific panel of markers, to be further explored in other clinical settings, namely at follow-up and detection of residual disease.

## 2. Results

### 2.1. Classical Serum Tumor Markers Have Limited Sensitivity for Detecting TGCTs

Clinicopathological features of the patient cohort are depicted in Table 1. The cohort consisted of 98 TGCTs, i.e., 21 SE and 77 NS, the majority being stage I (59.1%). Age at diagnosis was not significantly different between TGCT patients (median 31 years, IQR 26–36) and controls (median 37 years, IQR 27–48; *p* = 0.08).

**Figure 1:**
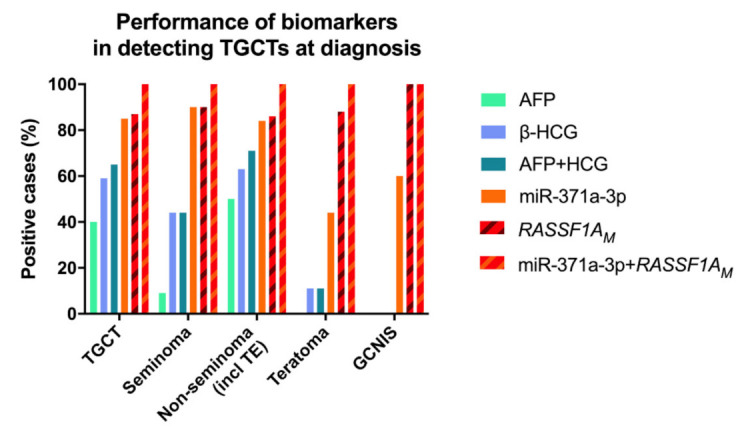
Performance of AFP, β-HCG, miR-371a-3p and *RASSF1A_M_* in detecting testicular germ cell tumor patients at time of diagnosis. Results are presented color-coded, referring to all testicular germ cell tumor patients (*n* = 98); all seminomas (*n* = 21); all non-seminomas (including teratomas, *n* = 77); all teratomas (*n* = 9); and all GCNIS-only patients (*n* = 5). Notice that both miR-371a-3p and *RASSF1A_M_* show better detection performance than the combination of both AFP and β-HCG, and that combination of the former two biomarkers results in 100% sensitivity for detecting TGCT patients, including those with teratoma and those with GCNIS-only. Abbreviations: AFP—alpha fetoprotein; β-HCG—human chorionic gonadotropin subunit β; GCNIS—germ cell neoplasia in situ; *RASSF1A_M_*—hypermethylated *RASSF1A*; TE—teratoma.

Pre-orchiectomy AFP and β-HCG levels were elevated above laboratory-set thresholds in 36/88 (40.9%) and 50/84 (59.5%) of TGCT patients (9.5% and 44.4% of SE; 50.7% and 63.6% of NS, respectively; Figure 1), showing the representative constitution of the cohort investigated [33]. At least one marker was elevated in 55/84 (65.5%) patients (Figure 1). 

**Figure 2:**
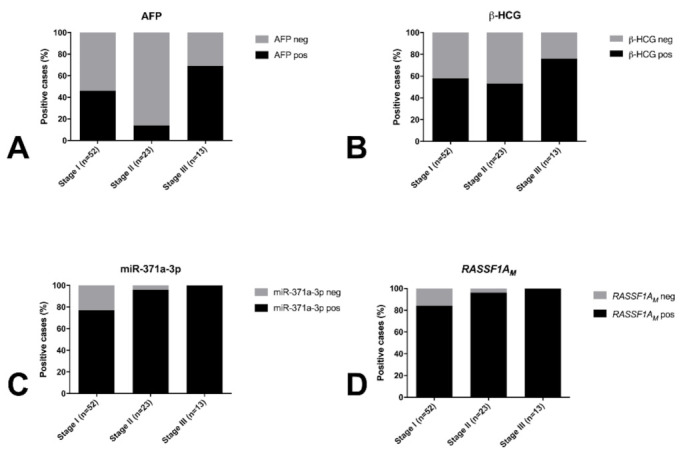
Proportion of positive cases for AFP (**A**), β-HCG (**B**), miR-371a-3p (**C**) and *RASSF1A_M_* (**D**) according to stage of disease. Abbreviations: AFP—alpha fetoprotein; β-HCG—human chorionic gonadotropin subunit β; *RASSF1A_M_*—hypermethylated *RASSF1A*.

One SE patient had remarkably elevated AFP levels and was treated as a NS. All TE samples were negative for AFP and only one positive for β-HCG. The two markers were negative in GCNIS cases. Positivity for both AFP and β-HCG significantly associated with higher disease stage (*p* = 0.001 and *p* = 0.009, respectively; Figure 2).

### 2.2. miR-371a-3p Testing Outperforms Classical Serum Markers in Detecting TGCTs at Diagnosis, but Is Limited in Detecting Teratoma

**Figure 3:**
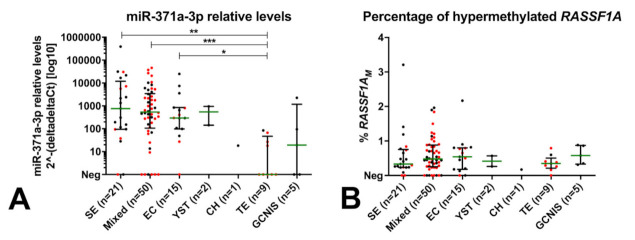
Relative levels of miR-371a-3p (**A**) and percentage of *RASSF1A_M_* (**B**) across histological subtypes. Negative samples (as by the defined cutoffs) are plotted at the *xx*-axis (Neg). Stage I patients are highlighted in red. miR-371a-3p relative levels are normalized to miR-30b-5p and plotted in log10 format for readability. Bars represent median and interquartile range. * *p* < 0.05; ** *p* < 0.01; *** *p* < 0.001 (adjusted for multiple comparisons). Abbreviations: CH—choriocarcinoma; EC—embryonal carcinoma; GCNIS—germ cell neoplasia in situ; SE—seminoma; *RASSF1A_M_*—hypermethylated *RASSF1A*; TE—teratoma; YST—yolk sac tumor.

Using the defined cutoff, all control samples were miR-371a-3p negative (i.e., no false-positive results occurred), as were all the non-GCTs (Leydig and Sertoli cell tumors). The miR-371a-3p test detected 84/98 TGCT patients (19/21, 90.5% SE and 65/77, 84.4% NS; Figure 1, Table 2) and 3/5 GCNIS-only patients; only 14 TGCTs were regarded as negative, five of them being pure TE, seven being stage I, one being stage II and one having no staging available. Overall performance of the test in discriminating TGCT patients from young-adult male healthy controls is shown in Supplementary Table S1. Overall sensitivity of detection of TGCTs was superior if TE samples (*n* = 9) were excluded and only non-TE histologies were considered (Supplementary Table S1). miR-371a-3p testing outperformed the combination of classical serum markers AFP or β-HCG in detecting TGCTs at diagnosis, especially for non-TE cases (Figure 1, Supplementary Table S1). Positivity for miR-371a-3p significantly associated with higher disease stage (*p* < 0.0001; Figure 2C). Relative levels of miR-371a-3p were significantly lower in TE compared to SE, mixed tumors and pure embryonal carcinoma (excluding pure yolk sac tumor, pure choriocarcinoma and GCNIS from the analysis, due to low numbers per category; adjusted *p*-values 0.0014, 0.0010 and 0.0256, respectively; Figure 3A).

### 2.3. Hypermethylated RASSF1A (RASSF1A_M_) Is Detected in GCT Cell Lines, Conditioned Medium and TGCT Tissue Samples

**Figure 4:**
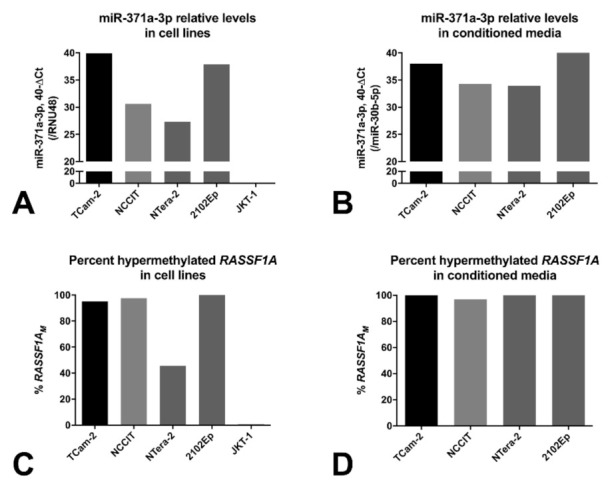
Relative levels of miR-371a-3p in cell lines (**A**) and in conditioned media (**B**), and percentage of *RASSF1A_M_* in cell lines (**C**) and conditioned media (**D**). miR-371a-3p relative levels are plotted in 40-ΔCt format for readability (normalized to RNU48 or miR-30b-5p in cell lines and conditioned media, respectively). Abbreviations: *RASSF1A_M_*—hypermethylated *RASSF1A*.

**Figure 5:**
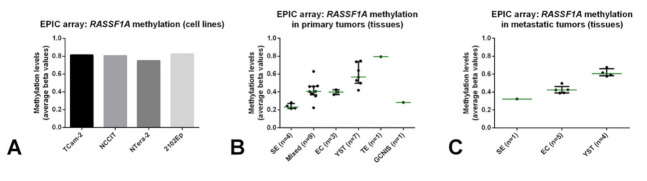
Estimated methylation levels for *RASSF1A* promoter derived from EPIC array analyses on cell lines (**A**), primary tumor tissues (**B**) and metastatic tumor tissues (**C**). Average beta values are plotted. Notice that the single teratoma sample has the highest methylation levels. Abbreviations: EC—embryonal carcinoma; GCNIS—germ cell neoplasia in situ; SE—seminoma; TE—teratoma; YST—yolk sac tumor.

The microRNA profiling analyses showed, as expected, that the miR-371a-3p is detected in all GCT cell lines (contrarily to the non-GCT JKT-1 cell line; Figure 4A, normalized to RNU48), and is also secreted into the medium (Figure 4B, normalized to miR-30b-5p), as we previously demonstrated [34]. Following our observations of detection of *RASSF1A* promoter methylation in TGCT tissue samples [29], we also performed methylation profiling with EPIC array on four GCT cell lines and 35 TGCT tissue samples (25 primary and ten metastatic). Average estimated methylation values for the promoter region of *RASSF1A* gene were above 0.75 for all the four GCT cell lines (Figure 5A). All patient samples (both primary and metastatic tumors) showed methylation levels above 0.20, but lower compared to all four GCT cell lines (Figure 5B,C). TE and yolk sac tumors displayed the highest methylation levels compared to seminoma, embryonal carcinoma and mixed tumors. The single GCNIS sample was similar to the seminomas.

Based on these data, we performed ddPCR to detect *RASSF1A_M_* in the various GCT representative cell lines and matched conditioned medium. *RASSF1A_M_* was detected in all GCT cell lines (while the non-GCT cell line JKT-1 was negative, in line with the cellular fraction, Figure 4C) and their conditioned medium (over 95% *RASSF1A_M_*, Figure 4D). This in vitro data (along with a previous study on clinical samples [30]) suggested the possibility of detecting *RASSF1A_M_* in liquid-biopsy samples from GCT patients.

### 2.4. RASSF1A_M_ Detection in Serum by ddPCR Is Sensitive and Specific for TGCT Detection at Diagnosis, including the Teratoma Subtype

Using the defined cutoff for *RASSF1A_M_*, a concentration of at least 0.36 copies/μL based on *RASSF1A_M_* background in control samples, *RASSF1A_M_* detected 85/98 (86.7%) TGCT patients (19/21, 90.5% SE and 66/77, 85.7% NS; Figure 1 striped red/brown bars, Table 2) and all five GCNIS samples. Only 13 tumors were regarded as negative (eight mixed tumors, two SE, two embryonal carcinomas and one TE, eight of these being stage I disease). Importantly, all but one TE samples were identified as positive, as well as the six non-GCT samples (Sertoli and Leydig cell tumors). It should be noted that in serum tested from healthy controls, there was no significant correlation between the concentration of *RASSF1A_M_* and total cfDNA (ACTB) (r = −0.09, *p* = 0.63).

The percentage of *RASSF1A_M_* in cfDNA isolated from serum of TGCT patients varied between 0% and 3.21%, with no significant differences among the various histological subtypes (Figure 3B). Overall, using the set cutoff, *RASSF1A_M_* detected TGCT patients with 86.7% sensitivity (Supplementary Table S1). This outperformed the classical serum markers in TGCT detection (Figure 1 striped red/brown bars). The proportion of *RASSF1A_M_* positive patients increased significantly with higher disease stages (*p* < 0.0001; Figure 2D).

Importantly, all the 14 TGCT cases scored as miR-371a-3p negative were *RASSF1A_M_* positive, resulting in the detection of 100% of patient samples when both markers were combined, with no false-positive results in the pool of control subjects (Figure 1 striped red/orange bars).

## 3. Discussion

AFP and β-HCG are the current serum biomarkers used for the clinical handling of TGCT patients. However, there are clinically important limitations: they are elevated in only about 50–60% of patients at time of diagnosis [34] (similar to our cohort, Figure 1) and are highly dependent on the histological composition, with subtypes such as SE and TE frequently not demonstrating elevations of these markers [13,35,36]. They may also be elevated in individuals with non-germ cell malignancies or in other medical conditions [34]. Better biomarkers are needed to be introduced in the clinic, complementing these classical markers and filling the diagnostic gaps. In the last decade, the most informative biomarker has been the miR-371a-3p; various large studies, retrospective and more recently also prospective [17,20], have consistently demonstrated the value of this microRNA as a biomarker of disease, overpowering the combination of the classical markers [37], and the present study constitutes further independent validation of these findings (Figure 1). The high sensitivity and specificity of miR-371a-3p at diagnosis (consistently over 85% [34]; 85.7% and 100% respectively in our study, Supplementary Table S1) is related to its expression pattern during embryogenesis, a process that (T)GCTs closely resemble [9,10]; also, the high relative levels detected in several body fluids is illustrated by the various GCT cell lines, which actively secrete this microRNA into the culture medium [33] (Figure 4A,B). The exact targets and mechanisms regulated by miR-371a-3p are still under-explored [38]; however, miR-371a-3p is for now the best and most versatile non-invasive biomarker (actually the most informative player within the miR-371-373 cluster [39]) for TGCT patients, useful for diagnosis, follow-up, prediction of response to therapy and prediction of viable disease after chemotherapy. The main critic directed to miR-371a-3p has been the lower performance in detecting TE [21] (and also GCNIS [40]) compared to other histological subtypes, not fulfilling the requirements of an informative and reliable marker in these contexts; this was also evidenced by the present study, with less than half of TE being detected and only 3/5 GCNIS (Figure 1, Table 2), in line with earlier findings [41]. This is a clinical challenge, since TEs commonly present as metastatic masses after chemotherapy, and are no longer susceptible to systemic treatments, requiring often technically difficult surgical excision that is only performed in reference centers [23,24]. Clinical decision on how to perform follow-up and how to approach these patients relies on measuring AFP/β-HCG and on interpretation of computed tomography scans, which have an even more limited detection ability in this context [42]. Identification of a novel biomarker to combine with miR-371a-3p and the current clinical tools would constitute a major advance in the field, namely for the detection of relapses in the form of metastatic disease.

**Figure 6:**
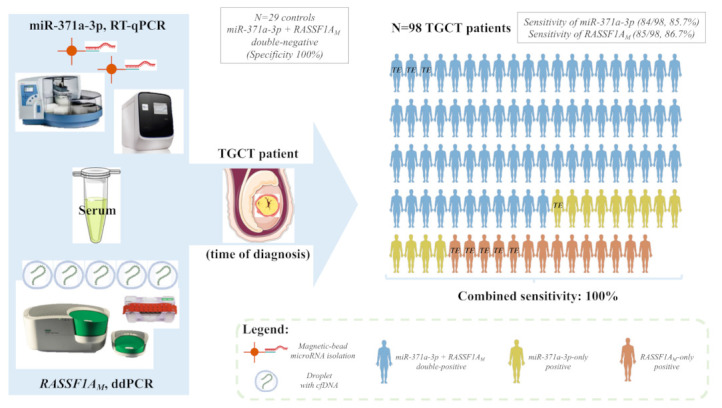
Illustrative overview of the combined pipeline. Serum samples of 98 TGCT patients and 29 healthy male blood donors were submitted to miR-371a-3p quantification by real-time quantitative PCR and to *RASSF1A_M_* quantification via ddPCR. Each male patient figure represents one of the TGCT patients in the study. The color code identifies if the patient was diagnosed because of positivity for both markers (blue), positivity for miR-371a-3p only (yellow) or positivity for RASSF1A only (orange). The patients with pure teratoma are highlighted by “TE”. The combined pipeline detected all TGCT patients (including those with pure teratoma) while all tested controls were negative, resulting in a combined sensitivity and specificity of 100%. Abbreviations: ddPCR—droplet digital PCR; cfDNA—cell-free DNA; *RASSF1A_M_*—hypermethylated *RASSF1A*; RT-qPCR—real-time quantitative PCR; TE—teratoma; TGCT—testicular germ cell tumor.

*RASSF1A* is a “pan-cancer” gene, in a sense that it plays various roles in oncogenesis (and in other patho-physiological processes), being hypermethylated in a wide range of adult and childhood neoplasms, frequently acting as an epigenetic sensor, anticipating neoplastic cell transformation [27]. Treatment of (T)GCT cell lines with the demethylating agent 5-aza-2′-deoxycytidine led to an increase in transcript levels of *RASSF1A*, evidencing the epigenetic silencing of *RASSF1A* also in this tumor model [43]. Moreover, methylation of *RASSF1A* promoter was also shown in TGCT tissue samples, including in the TE subtype, suggesting that this marker is also informative for all histological subtypes [29,44,45]. *RASSF1A* interferes with several cancer networks and pathways that integrate the general hallmarks of cancer, hence being frequently an informative biomarker for many cancers. In particular, *RASSF1A* has also been implicated in regulation of stemness, cell cycle and apoptosis (cell fate decisions), which are mechanisms highly involved in GCTs genesis, progression and response to cisplatin-based therapy [46]. Indeed, our methylation profiling analyses with EPIC array showed methylation of *RASSF1A* promoter in (T)GCT cell lines and in tumor tissues, with TE exhibiting even the highest methylation levels (Figure 5). The lower percentage of *RASSF1A_M_* detection in NTera-2 cells when compared to the conditioned medium may be due to secretion-related reasons. However, data on its use as a liquid biopsy biomarker of these tumors is scarce. Ellinger et al. [30] provided supportive data on the detection of *RASSF1A_M_* in serum samples of TGCT patients. The study, which shows similarities to ours (use of serum of TGCT patients at diagnosis, use of a methylation-sensitive restriction enzyme) was based on quantitative methylation-specific PCR and assessed 73 TGCT patients, but detected only 47% TGCTs (42% SE and 51% of NS). This relevant study seems to indicate that an improvement in test sensitivity is desirable. In our study, we addressed this by performing ddPCR and have also included more patient samples (*n* = 98 TGCT patients). This technique is recently emerging as a novel methodology with high sensitivity, which is particularly desired in liquid biopsies [47,48]. Moreover, to overcome further DNA degradation introduced by bisulfite treatment [49] (for specifically detecting methylated CpGs), we made use of a methylation-specific restriction enzyme. Because there was no correlation between the concentration or *RASSF1A_M_* and total cfDNA in serum samples of healthy controls, we proceeded using the single methylation-specific restriction enzyme digestion. All of this in combination allowed us to achieve the highest performance; indeed, using the set threshold based on the controls, *RASSF1A_M_* allowed for a diagnostic sensitivity of 86.7%. *RASSF1A_M_* showed a similar performance to miR-371a-3p (Figure 1 and Supplementary Table S1), the most informative biomarker to date. It also outperformed the combination of the classical serum markers and, additionally, was less dependent on histology, detecting 8/9 of the TEs and also samples from GCNIS-only patients (i.e., without an invasive TGCT component), illustrating the high sensitivity of our assay. In fact, all 14 tumors regarded as negative by miR-371a-3p testing were detected by *RASSF1A_M_*, in practice resulting in a 100% sensitivity when both markers were combined in this independent and representative TGCT cohort (Figure 6). Detection of *RASSF1A_M_* in non-GCTs of the testis (Sertoli and Leydig cell tumors) is not surprising given the broad role of the *RASSF1A_M_* in tumorigenesis [27]. However, non-GCTs of the testis (which are miR-371a-3p negative, like most TE) are almost invariably benign and treated by orchiectomy alone, not representing the same clinical challenges as TGCTs, namely for follow-up. Moreover, like for miR-371a-3p, *RASSF1A_M_* appears to relate to tumor burden, since an overall increase in positive cases was seen with increasing stage, in line with results from Costa et al. [29] in tissue samples. Classical tumor markers also followed the same tendency, with the decrease in proportion of positive cases for AFP in stage II being explained by the high proportion of SE (which are AFP-negative) in the stage II group (56.5%).

One of the limitations of our work is the limited amount of serum volume available for testing, as well as the use of serum instead of plasma patient samples for cfDNA analyses, since tumor-specific cfDNA is diluted by high concentrations of non-specific genomic DNA, released during the clotting process of white blood cells in the collection tube [50]. However, we made use of a representative cohort of serum samples collected throughout the Western part of the Netherlands, with clinical and histological data available and revised by an experienced pathologist. Also, although the number of tested samples could be further increased, we have applied a strict cutoff of positivity for both biomarkers, meaning that no false positive results are found. This way, the magnitude of the difference between the distribution of controls and patients is large, translating into the need for smaller sample sizes for achieving the desired statistical power of the observations.

## 4. Materials and Methods

### 4.1. Serum Samples

A total of 109 pre-orchiectomy serum samples from 109 individual patients were included in the study (98 TGCTs, 5 GCNIS-only, 4 Leydig cell tumors and 2 Sertoli cell tumors). Blood samples were collected right before surgery, between 2000–2018 in several centers across the Netherlands, processed and stored at −80 °C. All orchiectomy specimens were histologically confirmed and characterized by a TGCT-dedicated pathologist.

A total of 29 healthy young adult male serum samples (controls) were collected from Sanquin (Amsterdam and Rotterdam), and included in the analyses for setting thresholds of positivity (see details below).

Use of patient samples (archival samples only) was approved for research by the Medical Ethical Committee of the EMC (The Netherlands), permit no. 02.981.

### 4.2. Cell Lines and Tissue Samples

The following cell lines and conditioned medium (TCam-2, NTera-2, NCCIT, 2102Ep and JKT-1) were included, previously characterized by us [51] and cultured as described [33,52]. Thirty-five TGCT tissue samples originating from an independent patient cohort were also included and submitted to Illumina’s EPIC array [53].

### 4.3. Cell-Free DNA Isolation

Between 200 and 1000 µL of serum and 5 mL conditioned media were available for cfDNA isolation, which was performed with the Quick-cfDNA Serum & Plasma kit (Zymo Research, Irvine, CA, USA), according to manufacturer’s protocol. 

### 4.4. Genomic DNA Extraction from Cell Lines and Tissues and EPIC Array

Genomic DNA was extracted from cell lines using the DNeasy Blood and Tissue Kit (Qiagen, Venlo, The Netherlands) according to manufacturer’s instructions, as described before [54].

To test the hypermethylation of *RASSF1A* promotor region within the different histological tumor subtypes, we performed DNA extraction and bisulfite treatment of cell lines and 35 tumor tissue samples, followed by methylation profiling with the EPIC array (as performed in [53]). Minimal tumor percentage in each sample was 75%. Average estimated methylation levels (beta-values) for the *RASSF1A* promoter region of the several samples were plotted.

### 4.5. ddPCR for Hypermethylated RASSF1A

ddPCR for *RASSF1A_M_* was performed by a novel method [55]. To distinguish between methylated and unmethylated *RASSF1A*, every sample was subjected to two different ddPCR reactions: one with the methylation-specific restriction enzyme BstUI, and one without it; all remaining conditions were identical among the two reactions. Reactions with enzyme were performed in duplicate and those without the enzyme in single. Reaction mixes for ddPCR were prepared to a final volume of 22 µL using 11 µL ddPCR Supermix for probes (no dUTP) (Bio-Rad Laboratories, Hercules, CA, USA), 1 µL of *RASSF1A* and ACTB-1 assays (final concentration of 900 nM of each primer and 250 nM of each probe), 0.5 µL of ACTB-2 assay (final concentration of 450 nM of each primer and 125 nM of probe), 7 µL of DNA eluate, 1 µL BstUI (New England BioLabs, Ipswich, MA, USA) and 0.5 µL/1.5 µL H2O for reactions with/without enzyme, respectively. Droplets were generated using the QX200™ Droplet Generator (Bio-Rad). Incubation and thermal cycling was performed using the C1000 Touch Thermal Cycler (Bio-Rad), with the following program: 60 °C for 60 min, 95 °C for 10 min; 40 cycles of 94 °C for 30 s then 59 °C for 1 min; 98 °C for 10 min; 4 °C hold. Following PCR, droplets were read and quantified using the QX200 Droplet reader and QuantaSoft™ Software (version 1.7.4, Bio-Rad Laboratories). Amplitude thresholds were manually set by the operator on the basis of positive and negative droplet amplitudes produced by no template controls, neuroblastoma cell line IMR32 (100% *RASSF1A_M_*—positive control) and genomic DNA from peripheral blood mononuclear cells from a healthy donor pool (negative controls). ACTB-1 was added to control for cfDNA input, since this amplicon is unaffected by BstUI. ACTB-2 was added to control for BstUI performance, since this amplicon will be digested by the enzyme, resulting in no amplification in reactions with BstUI. Primer and probe sequences for RASSF1A and ACTB-2 have been described before by O’Brien et al. [56]. Primers and probes used for *RASSF1A*, ACTB-1 and ACTB-2 are listed in Supplementary Table S2. 

A patient sample was scored positive if the concentration of *RASSF1A_M_* was above the set threshold of 0.36 copies/μL, based on the mean + 3 × SD of the *RASSF1A_M_* concentration in the set of 29 healthy controls. If a sample was scored positive, the percentage of *RASSF1A_M_* was calculated as (ratio (*RASSF1A*/ACTB with enzyme)/(*RASSF1A*/ACTB without enzyme) × 100%.

### 4.6. MicroRNA Isolation from Serum, Targeted Analyses and Quality Control

MicroRNAs were isolated from 50 µL serum using the ampTSmiR test (magnetic bead-based isolation using the TaqMan^®^ miRNA ABC Purification Bead Kit and the KingFisher Flex with 96 KF Head, Thermo Fisher Scientific, Waltham, MA, USA), as described before [33,41,57]. MicroRNA isolation was followed by cDNA synthesis, a 12-cycle pre-amplification step, and real-time quantitative polymerase chain reaction (RT-qPCR) for each target: ath-miR159a (assay 000338), hsa-miR-30b-5p (assay 000602) and hsa-miR-371a-3p (assay 002124) (Thermo Fisher Scientific). The reaction was run in the QuantStudio 12K Flex Real-Time PCR System (Thermo Fisher Scientific). For cell lines and conditioned medium, microRNA profiling was performed on bead-captured microRNAs using TaqMan Low-Density Array (TLDA) Cards (detailed protocol in (28)).

The non-human microRNA spike-in (ath-miR-159a) was added in a fixed amount during microRNA isolation, as quality control, as described (28). To assure RT-qPCR efficiency and inter-plate comparability, serial dilutions (1:8) of cDNA from the seminoma-like cell line TCam-2 (26) were included as positive controls. A no template control was included as negative control for every assay.

The miR-371a-3p levels were relatively quantified according to the 2^−ΔΔCT^ method (normalized to the endogenous reference miR-30b-5p). Results were calibrated to the median ΔCt of controls, as previously reported [22,33,58], using specifically 21 controls with remaining serum sample after *RASSF1A* studies. The raw deltaCt values are provided as Supplementary File S1. miR-30b-5p has been the most widely used housekeeping microRNA for use in TGCT studies, also being shown to be less influenced by external factors such as hemolysis in dedicated technical investigations using the same pipeline [33]. There is no validated universal cutoff for reporting miR-371a-3p (as discussed in [37]). To avoid false positivity, a strict threshold of a minimum of 3 Ct below the median ΔCt of the control samples was set as cutoff for considering the miR-371a-3p test as “positive”, adapting the guidelines reported for minimal residual disease quantification in [59]. False positive results in the TGCT population would result in overtreatment of young males, one of the major problems in the field at the moment. 

### 4.7. Statistical Analyses

Data was represented as median and interquartile range (IQR). Associations between categorical variables were assessed using the Chi-square test or Cochran-Armitage test for trend, as appropriate. Continuous variables were assessed using non-parametric tests Mann-Whitney U, Kruskal Wallis and Spearman correlation, as appropriate. *p*-values were adjusted to multiple comparisons by the Dunn’s test. Statistical significance was set at *p* < 0.05.

## 5. Conclusions

In conclusion, our sensitive and specific assay for detecting *RASSF1A_M_* may fill in the gaps left open by the miR-371a-3p and classical markers. The combination of *RASSF1A_M_* and miR-371a-3p has the potential to lead to better clinical handling of TGCT patients.

## Figures and Tables

**Table 1:** Clinicopathological features of the pre-orchiectomy TGCT sample cohort.

Variables	TGCTs (*n* = 98)
Age [years (median, interquartile range)]	31 (26–36)
Histologic subtypes (*n*, %)	
Pure seminoma *	21/98 (21.4)
Pure embryonal carcinoma	15/98 (15.3)
Pure postpubertal-type yolk sac tumor	2/98 (2.1)
Pure choriocarcinoma	1/98 (1.0)
Pure postpubertal-type teratoma	9/98 (9.2)
Mixed tumor	49/98 (50.0)
Synchronous bilateral tumor (mixed tumor + teratoma)	1/98 (1.0)
Tumor size [cm (median, interquartile range)]	3.5 (2.5–5.1)
Stage (*n*, %)	
I	52/88 (59.1)
II	23/88 (26.1)
III	13/88 (14.8)
Rete testis invasion (*n*, %)	
Absent	56/91 (61.5)
Present	35/91 (38.5)
Vascular invasion (*n*, %)	
Absent	48/93 (51.6)
Present	45/93 (48.4)
**Variables**	**Others (*n* = 11)**
Histologic subtypes (*n*, %)	
GCNIS	5/11 (45.5)
Leydig cell tumor	4/11 (36.3)
Sertoli cell tumor	2/11 (18.2)

* includes one seminoma patient with very high levels of alpha fetoprotein, treated as a non-seminoma. Abbreviations: GCNIS—germ cell neoplasia in situ; TGCT—testicular germ cell tumor.

**Table 2:** Detection of the various TGCT histological subtypes using the miR-371a-3p and RASSF1AM as serum biomarkers.

Variables	miR-371a-3p Positivity (*n*, %)
All TGCTs	84/98 (85.7%)
Pure seminoma *	19/21 (90.5%)
Pure embryonal carcinoma	14/15 (93.3%)
Pure postpubertal-type yolk sac tumor	2/2 (100%)
Pure choriocarcinoma	1/1 (100%)
Pure postpubertal-type teratoma	4/9 (44.4%)
Mixed tumor ^#^	44/50 (88.0%)
GCNIS	3/5 (60.0%)
**Variables**	***RASSF1A_M_* Positivity (*n*, %)**
All TGCTs	85/98 (86.7%)
Pure seminoma *	19/21 (90.5%)
Pure embryonal carcinoma	13/15 (86.7%)
Pure postpubertal-type yolk sac tumor	2/2 (100%)
Pure choriocarcinoma	1/1 (100%)
Pure postpubertal-type teratoma	8/9 (88.9%)
Mixed tumor ^#^	42/50 (84.0%)
GCNIS	5/5 (100%)

* includes one patient with very high AFP levels, treated as a non-seminoma. ^#^ includes the patient with a synchronous bilateral tumor (mixed tumor + teratoma). Abbreviations: GCNIS—germ cell neoplasia in situ; *RASSF1A_M_*—hypermethylated *RASSF1A*; TGCT—testicular germ cell tumor.

## Data Availability

All data produced in the study is available within the manuscript and its supplementary files.

## Supplementary Materials

The following are available online at https://www.mdpi.com/article/10.3390/cancers13205228/s1. Supplementary Table S1: Performance on discriminating TGCTs from controls using the miR-371a-3p and *RASSF1A_M_* as serum biomarkers. Supplementary Table S2: Primers and probe sequences; Supplementary File S1: DeltaCt values for miR-371a-3p in TGCTs and controls.
