# Supplementary material for: Combining Hypermethylated RASSF1A Detection Using ddPCR with miR-371a-3p Testing: An Improved Panel of Liquid Biopsy Biomarkers for Testicular Germ Cell Tumor Patients
Source: Cancers (Basel). 2021 Oct 18;13(20):5228. doi: 10.3390/cancers13205228 (PMC8534014; doi:10.3390/cancers13205228)
Supplement: Supplementary file 1 [file cancers-13-05228-s001.zip › cancers-1363090-supplementary-final.pdf]

cancers

MDPI

Article

Combining Hypermethylated *RASSF1A* Detection Using ddPCR with miR-371a-3p Testing: An Improved Panel of Liquid Biopsy Biomarkers for Testicular Germ Cell Tumor Patients

João Lobo, Lieke M.J. van Zogchel, Mohammed G. Nuru, Ad J.M. Gillis, C. Ellen van der Schoot, Godelieve A.M. Tytgat and Leendert H.J. Looijenga

Supplementary Methods

Table S1. Performance on discriminating TGCTs from controls using the miR-371a-3p and *RASSF1A*M as serum biomarkers.

Serum biomarker	Context	Sensitivity (%)	Specificity (%)	PPV (%)	NPV (%)
miR-371a-3p	All TGCTs	85.7	100	100	60
	All TGCTs (excl. TE)	89.9	100	100	70
*RASSF1A*M	All TGCTs	86.7	100	100	69.1
miR-371a-3p + *RASSF1A*M	All TGCTs	100	100	100	100

Abbreviations: PPV – positive predictive value; NPV – negative predictive value; *RASSF1A*M – hypermethylated *RASSF1A*; TE – teratoma; TGCT – testicular germ cell tumor.

Table S2. Primers and probe sequences.

Target	Forward primer	Reverse primer	Probe	Amplicon length (bp)
RASSF1A	AGCCTGAGCTCATTGAGCTG	ACCAGCTGCCGTGTGG	5FAM/CCAACGCGCTGCGCAT/3MGBEc	129
ACTB-1	GTAAGGACAAGTTGGCCCCC	TGACTTTGTGGTGTGGCTGG	5HEX/TGCAGGGTT /ZEN/CACCCTCTGCTGCCCCCA /3IABkFQ	101
ACTB-2	GCGCCGTTCCGAAAGTT	CGGCGGATCGGCAAA	5HEX/ACCGCCGAGACCGCGTC/3MGBEc/	137
